# Time-lapse 3D image datasets of spruce tree wood enzymatic deconstruction

**DOI:** 10.1016/j.dib.2025.111618

**Published:** 2025-05-07

**Authors:** Solmaz Hossein Khani, Noah Remy, Khadidja Ould Amer, Berangère Lebas, Anouck Habrant, Grégoire Malandain, Gabriel Paës, Yassin Refahi

**Affiliations:** a Université de Reims-Champagne-Ardenne, INRAE, FARE, UMR A 614, Reims, France; b Université Côte d’Azur, Inria, CNRS, I3S, Nice, France

**Keywords:** Plant cell wall, Microscale, 4D(space + time) imaging, Image processing, Recalcitrance, Hydrolysis

## Abstract

The transition to use plant cell walls as an alternative to fossil carbon resources is important in the context of climate change. To achieve an economically viable plant cell wall transformation into biofuels and biomaterials, it is essential to better understand cell wall enzymatic deconstruction and overcome its recalcitrance to deconstruction. While identification of nanoscale markers of recalcitrance has been the focus of the majority of studies, quantitative investigation of cell wall hydrolysis at microscale, particularly the cell wall morphological parameters, remains relatively insufficiently addressed. This is mainly due to the lack of quantitative data on cell wall enzymatic deconstruction at microscale. Acquisition and processing of reliable microscale datasets are notoriously challenging; the sample needs to be kept at a constant temperature for efficient enzymatic hydrolysis and imaged over a considerable number of hours. Processing the acquired datasets to extract cell wall morphological parameters is also challenging due to cell wall deconstruction and deformations occurring during enzymatic hydrolysis. This becomes particularly challenging under high deconstruction conditions. The datasets presented here include time-lapse 3D images of highly deconstructed pretreated spruce wood acquired using fluorescence confocal microscopy, together with cell resolution segmentations of the acquired time-lapses. Along with this hydrolysis dataset, control time-lapse images of pretreated spruce wood samples acquired without adding enzymatic cocktail are also presented. The control dataset includes 6505 segmented and tracked cells. The hydrolysis dataset includes 6699 tracked cells at various stages of extensive deconstruction. Overall, these datasets provide a reliable and comprehensive set of time-lapse 3D images to study cell wall enzymatic deconstruction at cell and tissue scales, which can be used to better understand the microscale limiting factors of efficient transformation of plant biomass into sustainable products.

Specifications Table

**Table:** 

Subject	Biotechnology
Specific subject area	Plant biotechnology, Computational biotechnology, Enzymatic deconstruction, Recalcitrance, 4D (space + time) image processing
Type of data	Image Raw and processed
Data collection	Spruce (*Picea abies*) wood samples were pretreated using sodium chlorite to remove lignin and improve the enzymatic hydrolysis of cell walls. The samples were cut into transverse thin sections (40 µm) and were imaged hourly during hydrolysis over 24-hour using a fluorescence confocal microscope. Hydrolysis was carried out by an enzymatic cocktail mainly composed of cellulases and xylanases which was added to the sections. This yielded a set of time-lapse 3D images of cell wall hydrolysis. The control dataset was obtained by imaging pretreated sample cross sections into a buffer without enzymes. The time-lapse datasets were processed using the HydroTrack 4D segmentation and tracking pipeline.
Data source location	The data were acquired at FARE laboratory, Reims, France.
Data accessibility	Repository name: Zenodo Data identification number: 10.5281/zenodo.14547356 Direct URL to data: 10.5281/zenodo.14547356 Instructions for accessing these data: The time-lapses are provided as a single .zip file that can be downloaded using the above link and then unzipped. The structure of folders and files are described in the DATA DESCRIPTION section.
Related research article	Hossein Khani, S., Ould Amer, K., Remy, N., Lebas, B., Habrant, A., Faraj, A., Malandain, G., Paës, G., & Refahi, Y. (2025). A distinct autofluorescence distribution pattern marks enzymatic deconstruction of plant cell wall. *New Biotechnology, 88, 46-60.* doi: 10.1016/j.nbt.2025.04.001

## 1 Value of the Data

• For an economically viable transformation of plant cell wall into bioproducts, a deeper understanding of cell wall deconstruction is required. This requires dynamic data on the cell wall during enzymatic deconstruction.
• Although progress has been made, the cell wall enzymatic deconstruction remains insufficiently understood at microscale due to lack of quantitative data. The datasets presented here provide the first publicly available 4D (space + time) datasets of pretreated spruce wood enzymatic deconstruction.
• The datasets include time-lapse 3D images of cell wall enzymatic deconstruction using the natural cell wall autofluorescence along with control time-lapse images which can be used to develop mathematical models of cell wall hydrolysis.
• The datasets also include cell resolution segmentations of the time-lapse images. The impact and predictive capacity of cell scale quantitative analysis of deconstruction has been previously demonstrated.
• The datasets are reliable sources to develop deep learning algorithms for image processing. To the best of our knowledge, these are the first publicly available quantitative 4D cell wall deconstruction datasets. In general, reliable 4D datasets of different biological processes are not easily accessible to train algorithms.

## 2 Background

The transformation of plant cell walls into bio-based products, as alternatives to petroleum-based products, is increasingly important in the context of climate change [[1], [2], [3]]. A thorough understanding of cell wall deconstruction is essential to support an economically viable transition from fossil resources towards sustainable development. Although progress has been made [4], the cell wall enzymatic deconstruction at microscale remains under-examined, primarily due to a lack of quantitative datasets [5]. The collection of microscale datasets is a notoriously difficult task requiring meticulous efforts which involves imaging cell walls over several hours at a constant temperature while avoiding enzyme evaporation [6]. The cell wall resolution segmentation and tracking of the acquired datasets is also challenging but worthwhile as the pertinence and predictive capacity of investigating cell wall deconstruction at cell scale has been demonstrated [5]. The challenges of processing deconstruction datasets have been addressed by developing a robust pipeline, called HydroTrack [7] which enabled the generation of the datasets presented here. These datasets enable further analysis of cell wall deconstruction to draw new insights while promoting openness and transparency supporting reliability of the results. These datasets can also be used to develop mathematical models to investigate the underlying mechanisms of deconstruction.

## 3 Data Description

**Fig. 1:**
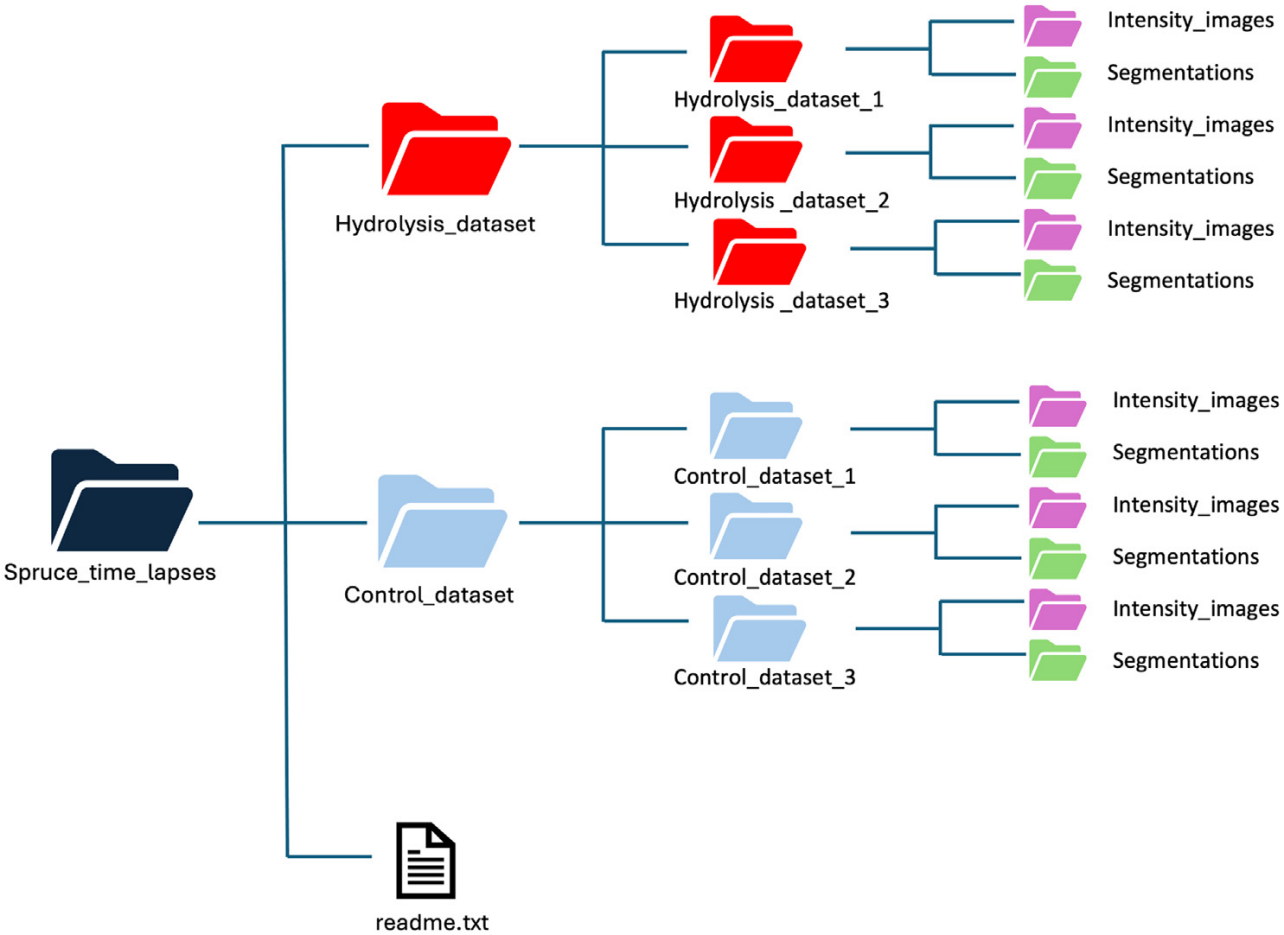
Data organisation. The datasets of hydrolysis and control time-lapses are organized into a hierarchy of folders and subfolders.

The datasets include both the collected hydrolysis and control time-lapse 3D images of pretreated spruce wood thin sections and their cell resolution segmentations (Fig. 1). The datasets are organized into a hierarchy of folders and subfolders [8]. The main folder named Spruce_time_lapses includes two subfolders, one for the hydrolysis dataset and one for the control dataset together with a descriptive text file named readme.txt with the following structure:

• **Spruce_time_lapses**
  This folder includes two folders for the hydrolysis and control time-lapse images and readme.txt file.

**1. Hydrolysis_dataset**

  This folder includes hydrolysis time-lapses collected in triplicates:

• Hydrolysis_dataset_1
• Hydrolysis_dataset_2
• Hydrolysis_dataset_3

**2. Control_dataset**

  This folder includes control time-lapses collected in triplicates:

• Control_dataset_1
• Control_dataset_2
• Control_dataset_3

Each of the folders containing time-lapses has two subfolders:

• **Intensity_images** includes twenty-five 3D images representing hourly acquisitions over 24 h. Each 3D image (confocal z-stack) is a .tif file named ***intensity*** followed by an index corresponding to the acquisition time (between 0 and 24, inclusive).
• **Segmentations** includes twenty-five segmented images representing cell resolution segmentation of the hourly acquisitions over 24 hours. Each 3D image is a .tif file named ***segmentation*** followed by an index corresponding to the acquisition time (between 0 and 24, inclusive).

The readme.txt file provides the above description which also includes the voxel size of the intensity and segmented images.

Each segmented image is a labelled image in which voxels of an individual cell are labelled with a unique label. These labels are consistent over time allowing cell identification and tracking over time.

## 4 Experimental Design, Materials and Methods

The datasets are collected using an experimental framework which involves sample preparation including sample pretreatment, sample cutting, and pre-incubation followed by enzyme solution preparation and incubation. Following the sample preparation, the sample is imaged during enzymatic hydrolysis using a fluorescence confocal microscope. The following provides detailed descriptions of these steps.

### 4.1 Sample pretreatment

Spruce wood samples were obtained by cutting blocks of 0.5 × 0.5 cm (in cross-section) and 1 cm (in length) from 2 cm cubes provided by INRAE Grand-Est. Sodium chlorite pretreatment was performed on fragments for delignification to enhance digestibility. Several 1h-baths at 70 °C using 1.25 g NaClO_2_, with 150 µL acetic acid in 40 mL of water as final volume were repeated, and associated with vacuum steps before each bath, in order to improve reagent diffusion in the sample. Acetyl bromide assay was performed to measure lignin content. Pretreated samples were conditioned (at 20 °C in desiccator).

### 4.2 Sample cutting

Following sample pretreatment, the 3 mm ending regions of the 1 cm axis of pretreated samples were used because the pretreatment affects the sample’s center and periphery differently. The sample was wrapped around in Parafilm, placed on the microtome’s head and moistened with distilled water. 40 µm sections were obtained using the microtome, equipped with a disposable blade.

### 4.3 Pre-incubation

Two sections were observed through a binocular light microscope to check that they were similar and unwounded. One of them was placed on a glass slide and moistened with a droplet of acetate buffer (50 mM, pH = 5). A gene frame was stuck on the sample and 60 µL of acetate buffer was added to the frame. Incubation was performed for 30 min.

### 4.4 Enzyme solution preparation and incubation

The second sample section was weighed with a precision balance. The stock enzyme was diluted with acetate buffer using the following equation:

$$V\;=\;\frac{V\left(flask\right)\;\times \;Mact\left(section\right)\;\times \;mass}{V\left(frame\right)\;\times \;Vact\left(stock\right)},$$

where

• *V* is the volume of stock enzyme to dilute (mL).
• *V(flask)* is the volume of the dilution flask (mL).
• *Mact(section)* is the mass activity wanted for the incubation (FPU/mg).
• *mass* is the mass of the section (mg).
• *V(frame)* is the volume of enzyme dilution to put in the frame (mL).
• *Vact(stock)* is the volumetric activity of the stock solution of the enzyme (FPU/mL).

**Fig. 2:**
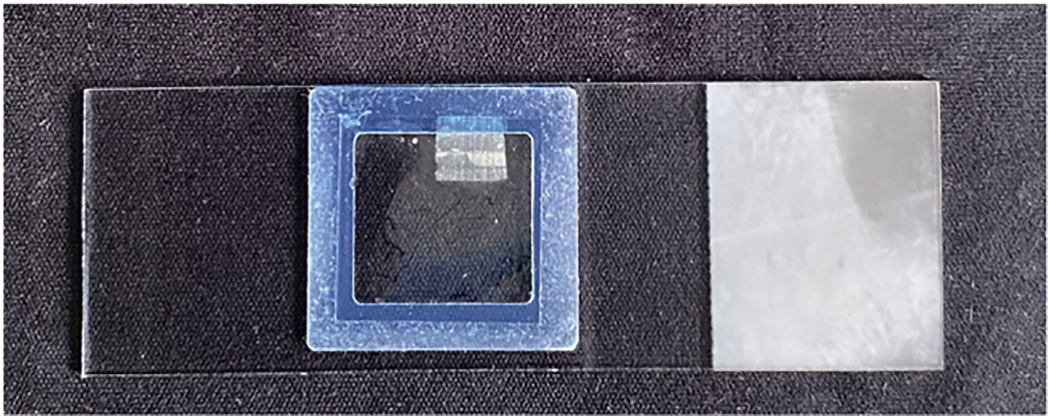
Glass slide with sample ready for microscopy.

When the 30-minute buffer incubation was over, the buffer was removed from the gene frame with filter paper. 60 µL of enzyme solution (or acetate buffer for control dataset) was added into the frame and the gene frame was sealed with a coverslip (see Fig. 2).

### 4.5 Fluorescence confocal imaging

The glass slide was put inside an incubation chamber with temperature stabilized at 50 °C. This setup was put on the microscope stage. A 3 × 3 grid of images of the sample was acquired, which allows a global mapping of the section. Time-lapse imaging was performed by acquiring 3D images over 24 hours with one-hour intervals.

### 4.6 Microscope acquisition parameters

A 405 nm excitation laser at 10 % power, with an emission detection range from 415 to 515 nm was used for acquisitions. Images were scanned at a speed of 4 µs/pixel with a resolution of 512 × 512 pixels, and the gain was set to 600 V. The confocal aperture was set to 50 µm. For preliminary mapping, an objective with a magnification of 4× with a zoom factor of 1× was used, while time-lapse imaging was performed using an objective with a magnification of 20× with a zoom factor of 2×. The Z-axis step size was set to 0.3 µm. Each acquisition involved a z-stack of 400 2D slices.

### 4.7 Identification of individual cells in time-lapse 3D images

To identify the individual cells in the collected time-lapse images, HydroTrack 4D image processing pipeline [4] was used. HydroTrack uses a combination of divide-and-conquer and temporal propagation of spatial information strategies to detect the individual cells in the time-lapse images. HydroTrack first segmented the pre-hydrolysis 3D image (as the first image of the time-lapse images) by first computing a convex hull of the sample image. The individual cells within the pre-hydrolysis image were identified by applying a watershed algorithm to the region inside the convex hull. This pre-hydrolysis segmentation was propagated to identify the corresponding cells in the subsequent time-lapse images. To do this, HydroTrack first divided the time lapse images into sequential clusters of a fixed number of successive images. Intra-cluster image registrations were performed to register the starting image of each cluster with the images within that cluster. The resulting transformations were then combined to propagate the pre-hydrolysis images and identify the individual cells over the course of hydrolysis. HydroTrack code is available via the FARE laboratory GitLab repository: https://gitlab.com/farelab/teamyr/publications/HydroTrack with an associated wiki which provides documentation and guidelines to support users in installing and using the software pipeline.

## Limitations

Not applicable.

## Ethics Statement

This work did not involve human subjects, animal experiments, or any data collected from social media platforms.

## CRediT Author Statement

**Solmaz Hossein Khani:** Conceptualization, Data curation, Formal analysis, Investigation, Methodology, Software, Validation, Visualization, Writing – original draft, Writing – review and editing. **Noah Remy:** Investigation, Methodology, Writing – original draft. **Khadidja Ould Amer:** Data curation, Methodology. **Berangère Lebas:** Investigation, Methodology, Writing – original draft. **Anouck Habrant:** Investigation, Methodology, Writing – original draft. **Grégoire Malandain:** Investigation, Methodology, Software, Writing – review and editing. **Gabriel Paës:** Conceptualization, Formal analysis, Funding acquisition, Investigation, Methodology, Project administration, Resources, Supervision, Validation, Writing – original draft, Writing – review and editing. **Yassin Refahi:** Conceptualization, Data curation, Formal analysis, Funding acquisition, Investigation, Methodology, Project administration, Resources, Software, Supervision, Validation, Visualization, Writing – original draft, Writing – review and editing.

## Data Availability

ZenodoTime-lapses of spruce tree wood hydrolysis (Original data).
